# Communication, on the Impropriety of Plugging Teeth with Two Kinds of Metals in the Same Mouth

**Published:** 1839-12

**Authors:** L. Mackall

**Affiliations:** Dentist, Baltimore.


### COMMUNICATION,

*On the impropriety of plugging teeth with two kinds of metals in the same mouth, by*

[ L. MACKALL, M.D. DENTIST, BALTIMORE. ]

There have recently appeared in one of our city papers, two communications, in which the opinion has been advanced, that two kinds of metals should never be used in plugging teeth. Every one has heard of the popular experiment, of placing silver on the top and zinc under the tongue, and making the edges touch to produce the peculiar metalic taste, caused by the galvanic action of the metals; but it is not generally known, even among the profession, that a constant galvanic action is kept up in the mouth, when more than one kind of metal is used in filling the teeth.

My attention was called to this subject some years since by my friend, Dr. Fenerden of this city, and from close observation since, as well as by a recollection of the cases that have occurred in my former practice, I am convinced that galvanism is often the cause of very extensive injury to the teeth. My present object is merely to call the attention of the profession to the subject, by reporting a few facts.

There is scarcely a day but every operator who has much practice will observe, that while he is filling a cavity with either gold or any other metal his patient will often complain of much tenderness when the plugger is brought in contact with the plug.—This pain or tenderness is always produced by a galvanic action between the instrument and the metal when any other metal is brought in contact with the plug; for example, a silver tooth pick. Wood, bone, or any other article but metal will not produce the same effect. Again, it will be generally observed, that teeth more frequently decay around the plugs, when two metals are used in the same mouth, than when all the cavities are filled with one kind; causing, I believe, invariably more irritation than other causes.

I very lately had a tooth filled with tin by my preceptor, others having been previously filled with gold. It was very much decayed, and gave me very considerable uneasiness during the operation, and continued to give me constant dull pain until it was removed, after which I could bear cotton to be pressed into the cavity with considerable force without any uneasiness. There was one symptom attending this case which is worthy of observation. There is an hereditary disposition to inflammation of the periosteum lining the fangs and alveolæ of my superior molar teeth on the left side, and while the tin remained in my tooth, (the *dens sapientiæ* of the lower jaw, same side,) there was a constant dull aching sensation in those parts.

The most distinctly marked case of galvanism in the mouth I have ever met with, was in a lady, on whose tooth I recently operated. I had filled several with tin for her, two or three years previous, and upon examination, I found the teeth had again decayed around the plugs, making it necessary to re-fill the cavities. While cleaning one of them out, she complained of much pain, which I attributed to inflammation of the bony structure only. When, however, I commenced re-filling it, the pain became so violent that I could not proceed, and upon trial, I found that by holding the instrument an inch or more from the tooth, I could at pleasure give her a violent galvanic shock. I filed the handle of a tooth brush to a suitable shape and filled the tooth readily without the slightest uneasiness. At the time, and even a week after, when a silver or steel instrument was held the same distance from the tooth, the same effects were produced. I observed that the exhalation of the breath increased the evolution of galvanism. If the patient kept her mouth open wide and held her breath, the instrument might be brought nearly in contact with the plug without producing any effect.—In conclusion, I may add, that these facts show how unscientific and injurious it must be to fill cavities with such "amalgams" and "pastes" as are sometimes strongly recommended by certificates from physicians, published by some dentists in the newspapers.

Note.—That the different metals exert and keep up a galvanic nervous influence in a very great number of instances, there can be no doubt; but how far, or to what extent they act upon each other when properly used, causing decay of the teeth or oxidation of the metals, is a question in my mind yet to be settled. Mr. Koecker, I believe, was the first that practiced the covering of the tender bone, or partially exposed nervous texture with lead, and then covering the lead with gold. I have seen teeth treated in this way twelve and fifteen years after the operation, and one tooth that has been filled twenty-one years, as the gentleman informed me last week, when both metals remained unchanged, except the inevitable wearing of the surface of the gold. The same practice was also followed to a great extent, in teeth that were not tender, by Mr. Newton, formerly of this city, some of whose operations in stopping teeth have never been surpassed by any dentist. I have very frequent opportunities of seeing teeth that he filled in this way ten, twelve and fifteen years ago, without being able to discover any other change in the metals than the wearing I have just spoken of. When the operation is perfectly performed, as it uniformly was by the gentlemen I have named, I am of opinion that no such results will follow when used in the same tooth or in several teeth in the same mouth; but where the cavity of the tooth, in the first place is imperfectly prepared—and where the metals are introduced in the awkward and clumsy manner that they are by three fourths of those who undertake to stop teeth, the worst consequences abovenamed will undoubtedly follow. All compound metals are decidedly pernicious; and the use of amalgams, pastes, &c., cannot, as above stated, be too strongly deprecated. In confirmation of the above, I here make a quotation from the work of the highly distinguished and accurate physiologist, Thomas Hare, Esq. F. L. S. F. R. C. S. L., published in 1820.

"Where a faulty tooth in the upper jaw had been stopped from its side with tin foil, the interstice between it and the adjoining tooth being quite inconsiderable, while the upper surface of a tooth not immediately beneath it in the lower jaw was stopped with the same metal, I have known a galvanic shock regularly communicated from one tooth to the other, whenever, by any accidental movement of the mouth, they came within a certain distance of each other, probably about a half an inch."

I understand there are those now in this city, who use the Crawcour Succedaneum and Amalgam of Silver and Mercury, our object being to correct errors in Dental Practice, we hope all such will discontinue its use, in justice both to the profession and the public; and also to save us the painful necessity of exposing their names. N. Y. ED.

